# Efficacy and Safety of Sofosbuvir and Ribavirin for Treating Chronic Hepatitis C, Genotype 3: Experience of a Tertiary Care Hospital at Karachi, Pakistan

**DOI:** 10.7759/cureus.4458

**Published:** 2019-04-14

**Authors:** Nazish Butt, Sehrish Reema, M Ali Khan, Amanullah Abbasi, Sehrish Butt, Mohammad M Khoso, Ali Akbar, Farhan Haleem, Ghulam B Mahesar, Anoshia Fahad, Talha Qureshi

**Affiliations:** 1 Gastroenterology, Jinnah Postgraduate Medical Centre, Karachi, PAK; 2 Internal Medicine, Jinnah Postgraduate Medical Centre, Karachi, PAK; 3 Internal Medicine, Dow University of Health Sciences, Karachi, PAK; 4 Biomedical and Biological Sciences, Dow University of Health Sciences, Karachi, PAK; 5 Internal Medicine, Jinnah Postgraduate Medical Center, Karachi, PAK

**Keywords:** safety, efficacy, sofosbuvir, genotype 3, chronic hepatitis c, ribavirin

## Abstract

Background

It is estimated that approximately 10 million individuals in Pakistan are infected with hepatitis C virus (HCV). Historically, it was very difficult not just to cure but even treat HCV as available options did not have desirable outcomes. However, the approval of directly acting antiviral (DAA) drugs has revolutionized treatment and management. These are specific proteases and polymerase inhibitors with profound capability for accomplishing elimination and overtime eradication of the virus.

Objective

The aim of this study was to evaluate the efficacy and safety of sofosbuvir (SOF) in combination with ribavirin (RIB) for the treatment of chronic hepatitis C virus with genotype 3.

Materials and methods

This prospective observational study was conducted at the gastroenterology section of Medical Unit IV, Jinnah Post-graduate Medical Center, Karachi and Medical Unit II, Dow University of Health Sciences, Ojha Campus, Karachi from January 2016 to December 2016. Patients aged 18 years or older of either gender having chronic active HCV infection as demonstrated by a positive Anti-HCV (ELISA) test and a qualitative polymerase chain reaction (PCR) analysis along with genotype analysis showing only type 3 were inducted into the study.

Treatment was initiated with either 12-week or 24-week regimen of SOF 400 mg once daily along with weight-adjusted RIB orally. Successful treatment was indicated by the elimination of the virus, i.e., undetectable viral load/levels by PCR qualitative analysis. Rapid virological response (RVR), end of treatment response (ETR), and sustained virological response (SVR) were defined as the undetectable viral load at four, 12, and 24 weeks, respectively.

Results

A total of 300 patients were inducted into the study, predominantly female (57%). The mean age of presentation was 41.14 ± 11.48, and most (70.33%) were treatment naïve. The mean alanine transaminase (ALT), aspartate aminotransferase (AST), and gamma-glutamyl transferase (GGT) levels at presentation were 41.89 ± 46.23 IU/l, 68.57 ± 83.62 IU/l, and 54.52 ± 77.57 IU/l, respectively.

ALT, AST, and GGT levels at 24 weeks were 33.84 ± 13.60 IU/l, 32.44 ± 16.16 IU/l, and 37.59 ± 22.41 IU/l, respectively, showing significant improvement. ETR was achieved in 99.1% (209) treatment-naïve patients and 98.9% (88) treatment-experienced patients. SVR rates were almost similar with 98% (208) achieving it in the treatment-naïve group and 96.6% (86) achieving it in the treatment-experienced group.

Conclusion

SOF in combination with RIB is safe and remarkably efficacious in the treatment of chronic HCV, genotype 3. Not only is this regimen associated with the elimination of viral replication but it also improved transaminase levels. Outcomes are rarely, if ever, affected by previous use of antiviral medications.

## Introduction

Hepatitis C Virus (HCV) affects about 170-200 million individuals worldwide and is the leading cause of chronic liver disease as well as decompensated liver disease in the developed world [1]. It is estimated that 10 million individuals in Pakistan are infected with HCV and this burden is expected to grow. Similarly, the prevalence HCV in the second largest province of Sindh is about 5%, afflicting approximately two million individuals [2-3].

HCV genotype-3 (GT-3) is the most common subtype in Pakistan. Previously, the only effective option for treating HCV infection was PEG-interferon. However, the outcomes with it were less than satisfactory, resulting in high morbidity and mortality especially with GT-3 [4]. Recently, directly acting antiviral (DAA) drugs have been approved by the Food and Drug Administration (FDA) of the United States. These have revolutionized the treatment of HCV [5-6].

Sofosbuvir (SOF) was the first DAA to be approved in December 2013 [7]. Since then, it has been in used in combination with other drugs, most notably ribavirin (RIB) with excellent results. DAAs have been so successful that now HCV is not only treatable but curable, and eradication of HCV is sought by the year 2030 [8].

## Materials and methods

This prospective observational study was conducted from January 2016 till December 2016 at Gastroenterology section of Medical Unit IV, Jinnah Postgraduate Medical Center, Karachi and Medical Unit II, Dow University of Health Sciences, Ojha Campus, Karachi. Written consent was taken from all patients. Patient confidentiality was made certain. All international standards were met, and the study was approved by the ethical board committee.

Inclusion criteria

A total of 300 patients were enrolled. Patients aged 18 years or older of either gender having chronic active HCV infection as demonstrated by a positive Anti-HCV (ELISA) test and a qualitative polymerase chain reaction (PCR) analysis along with genotype analysis showing type three only were inducted into the study.

Exclusion criteria

Patients with any of the following were ineligible for induction:

1. Cirrhotics with compensated or decompensated liver disease Cirrhosis was ruled out on the basis of history with clinical ( no ascites, encephalopathy, asterixis, coagulopathy or variceal bleed) and biochemical markers (Platelets, APRI score, etc.)

2. Patients who have already used SOF or any other DAAs for HCV

3. Patients treated for previous malignancy or going under chemotherapeutic regimens.

4. Active chronic hepatitis B Virus (HBV) infection, any other acute viral hepatitis (HBV carriers were eligible for induction)

5. Immunocompromised patients such as those with acquired immunodeficiency syndrome (AIDS).

6. Alcoholics and intravenous drug abusers

7. Patients with other liver disorders such as autoimmune hepatitis, Wilson’s disease, etc.

Primary outcome

The primary outcome was to evaluate the efficacy of the regimen used. Efficacy was shown by complete elimination of the virus from the serum as indicated by a negative/undetectable viral load via PCR qualitative analysis at four weeks since starting medication, i.e., rapid virological response (RVR), 24 weeks since starting medication i.e. end of treatment response (ETR) and six months post-treatment, i.e., sustained virological response (SVR).

Furthermore, RVR, ETR, and SVR were analyzed based on two categories. Treatment naïve group consisted of patients that had never taken any medication for HCV before and treatment experience group consisted of patients that had completed a course of any of previously available options to treat HCV. The treatment-experienced group had previously received PEG-interferon almost exclusively.

Secondary outcome

The secondary outcome was to record any adverse events (AEs), i.e., the safety of the drugs used, contributable to SOF and/or RIB use only during the treatment period. AEs could either be clinical, i.e., weakness, fever, etc., or laboratorial, e.g., reduction in hemoglobin (Hb), thrombocytopenia, hyperbilirubinemia, etc. AEs were monitored throughout the duration of treatment. 

Due to the vast array of AEs associated with RIB, only AEs that occurred during the study were analyzed and studied. Meticulous workup including nutritional, psychiatric, dermatologic, and orthopedic consults were done to ensure the AEs were solely due to the drugs used.

Drug Regimen

SOF inhibits the hepatitis C NS5B protein, therefore inhibiting viral replication. It is used in combination with multiple other drugs in treating HCV. RIB is a guanosine (ribonucleic) analog used to stop viral RNA synthesis and viral mRNA capping and therefore is a nucleoside inhibitor, while indicated for other purposes as well here it was used in the context of HCV only.

Tablet SOF was given at a dose of 400mg orally once (OD) daily in combination with tablet RIB 400mg orally twice (BID) daily. The treatment was continued for 24 weeks in all patients. 

Laboratorial Analysis

All patients were followed up with a complete blood count (CBC), liver function tests (LFTs), prothrombin time (PT), international normalized ratio (INR), urea with creatinine levels, and serum electrolyte levels at induction, four, 12 and 24 weeks after starting medications. Ultrasound abdomen was also performed at initial evaluation. 

Coinfection with HBV and human immune virus (HIV) were ruled out via their respective serologies. On rare occasions, where other liver pathologies were strongly suspected, work up with an autoimmune profile, 24-hour urinary copper, serum ceruloplasmin, and iron profile was carried out.

Data Analysis

In this study, non-probability convenient sampling technique was used. Data were analyzed using the Statistical Package for the Social Sciences (SPSS) version 21.0 software (SPSS Inc., Chicago, IL, USA). Data were presented as mean ± standard deviation (SD). The comparisons were performed with a paired *t*-test; chi-square test was used to evaluate the association of RVR, ETR, and SVR with the type of treatment, whereas repeated measures with ANOVA were applied to compare means of hematologic parameters measured at different time intervals. A *p*-value of <0.05 was considered statistically significant.

## Results

Demographic characteristics

The mean age of presentation was 41.14 ± 11.48 years. Patients were predominantly female (57%) and married (96%). Most patients presented were residents of Karachi (78%) and a high number (75%) did not have any comorbids. Demographics are summarized in Table 1.

**Table 1:** Demographic characteristics of patients

	N = 300 (%)
Marital Status	
Married	288 (96%)
Unmarried	12 (4%)
Gender	
Female	171 (57%)
Male	129 (43%)
Comorbids	
None	225(75%)
Hypertension	60 (20%)
Diabetes	12 (4%)
Others	3 (1%)
Age (Mean)	41.14 ± 11.48 years

Primary outcome

A high number of patients showed elimination of virus as evident by the excellent rates of RVR, ETR and SVR when treated with SOF+RIB. No correlation or significance was found between the treatment naïve or experienced groups. Overall only 6 (2%) patients out of 300 did not achieve SVR. The high rates of SVR demonstrated great efficacy on part of the drug regimen. Primary outcomes with comparative analysis of the two treatment groups are summarized in Table 2.

**Table 2:** Primary outcome and comparative analysis of the two treatment groups RVR: rapid virological response, ETR: end of treatment response, SVR: sustained virological response

Virological responses		Treatment naïve N = 211 (%)	Treatment experienced N = 89 (%)	p-value
RVR	Achieved	208(98.6)	84(94.4)	0.053
	Not Achieved	3(1.4)	5(5.6)	
ETR	Achieved	209(99.1)	88(98.9)	>0.999
	Not Achieved	2(0.9)	1(1.1)	
SVR	Achieved	208(98.6)	86(96.6)	0.366
	Not Achieved	3(1.4)	3(3.4)	

Secondary outcome

Adverse Events

Adverse events were far and few. The most common complains for the patients were fatigue (70.66%) and body ache (54.33%). Disease progression to decompensated cirrhosis was not seen at all. There was no incidence of melena, hemetemesis, hepatic encephalopathy, or development of ascites. Clinically, there were no signs of pallor (anemia) or shortness of breath in any of the patients (see below). Adverse events are summarized in Table 3.

**Table 3:** Adverse events experienced by the patients

	Treatment naïve N = 211(%)	Treatment experienced N = 89(%)
Fever	40 (19.0%)	46 (51.7%)
Dyspepsia	92 (43.6%)	54 (60.7%)
Fatigue	148 (70.1%)	64 (71.9%)
Rash	24 (11.4%)	6 (6.8%)
Abdominal Pain	41 (19.4%)	27(30.3%)
Bleeding Per Rectum	2 (0.9%)	3 (3.4%)
Body ache	120 (56.9%)	43 (48.3%)

Subgroup Analysis (Age Groups, Gender, and Comorbid)

Majority of the patients were females aged between 24-45 years for both treatment-experienced and naïve groups; 263 (87.33%) had no comorbidity. All factors favor improved outcomes. Subgroup analysis are summarized in Table 4. 

**Table 4:** Subgroup analysis

Characteristics	Treatment experienced	Treatment naïve
Age		
16-26	3 (2.1%)	25 (11.7%)
27-36	44 (31.8%)	67 (31.6%)
37-46	48 (34.7%)	54 (25.4%)
47-56	27 (19.5%)	46 (21.6%)
57-66	14 (10.1%)	19 (8.9%)
67-75	02 (1.4%)	0 (0)
Sex		
Male	48 (34.7%)	98 (46.2%)
Female	90 (65.2%)	114 (53.7%)
Co-morbid:		
Hypertension	28 (20.2%)	42 (19.8%)
DM	7 (5.7%)	5 (2.8%)
None	100 (72.4%)	163 (76.8%)

Laboratorial/Biochemical Parameters

The only concerning aspect was the mild decrease in hemoglobin level; this was found to be statistically significant. However, as discussed previously this did not have any clinical significance. All LFTs showed improvement over time. Analysis of labs is summarized in Table 5.

**Table 5:** Biochemical parameters with comparative analysis

Biochemical parameters	Baseline	1 Month	3 Months	6 Months	p-value
	Mean ± SD	Mean ± SD	Mean ± SD	Mean ± SD	
Hemoglobin (mg/dL)	12.19 ± 1.96	12.19 ± 1.96	11.89 ± 1.92	11.84 ± 1.87	<0.001
Mean Corpuscular Volume (fl)	84.44 ± 47.12	87.04 ± 65.30	87.47 ± 64.92	87.99 ± 65.10	0.05
Alanine Transaminase (units/liter)	68.57 ± 83.62	35.64 ± 22.33	36.35 ± 17.35	33.84 ± 13.60	<0.001
Aspartate Aminotransferase (units/liter)	41.89 ± 46.23	40.36 ± 45.97	36.93 ± 21.08	32.44 ± 16.16	<0.001
Alkaline Phosphatase (units/liter)	169.56 ± 99.41	165.41 ± 97.10	173.63 ± 89.72	161.21 ± 88.85	0.037
Gamma-Glutamyl Transferase (units/liter)	54.52 ± 77.57	50.08 ± 60.96	50.99 ± 61.43	37.59 ± 22.41	<0.001
Serum Albumin (g/dL)	3.93 ± 0.48	3.96 ± 0.50	3.94 ± 0.50	3.94 ± 0.51	0.739
Urea (mg/dL)	32.75 ± 20.93	34.31 ± 22.53	35.60 ± 23.77	33.32 ± 21.87	0.011
Creatinine (mg/dL)	0.88 ± 0.56	0.91 ± 0.58	0.84 ± 0.29	0.81 ± 0.28	0.005
Sodium (mEq/L)	134.80 ± 5.36	134.54 ± 4.91	134.68 ± 5.08	133.00 ± 14.79	0.05
Potassium (mEq/L)	4.02 ± 0.57	3.95 ± 0.56	3.99 ± 0.53	3.96 ± 0.51	0.02

## Discussion

HCV GT-3 was always cumbersome to treat, having substantially lower viral elimination rates, higher relapses, and overall poorer prognosis when treated with Peg-Interferon and RIB [9]. SVR rates for GT-3 were disappointing at best. This was particularly worrisome for Pakistan where GT-3 predominates [10]. Needless to say, this was associated with high morbidity and mortality.

The arrival of DAAs changed all of this. SOF led the way, with cure rates of ≥90 %. An exceptionally high SVR rate of 98% has been observed in our study, as reported by Zeuzem S. et al. [11]. There can be no doubt as to the efficacy of SOF in combination with RIB for treating chronic HCV. 

Out rate of SVR is impressive, but few things must be taken into consideration. First, irrespective of the treatment group (naïve vs. experienced), our patients did not have signs and symptoms of cirrhosis or decompensation. Second, most patients were young or middle-aged. Thirdly, there were few comorbidities in our patients. All three factors are good prognostic markers for viral elimination.

Alcohol use disorder (AUD) is rare in Pakistan and was absent among our patients to the best of our knowledge, this was again an important factor contributing to the impressive SVR. Low rates of non-alcoholic fatty liver disease (NAFLD) were seen in our study corresponding to low burden of diabetic patients, no doubt contributing to a high SVR.

A vast array of AEs was seen but none were troublesome enough for patients to drop out, affect their compliance or affect their quality of life. Furthermore not all AEs such as dyspepsia, body ache and bleeding per rectum could be attributed solely to the drug regimen despite comprehensive workup (see methods). Fatigue, the most common AE that could clearly be attributed to SOF did not affect the quality of life significantly and was easily overcome.

Fever was documented, mostly low to moderate grade and resolved over time, correlating with viral elimination. Abdominal pain was vaguely localized but consistently involved the right upper quadrant and showed resolution with the initiation of treatment. Significant weight loss was not seen at all.

The one major concern was a mild reduction in the Hb levels. This was most probably due to RIB. The MCV demonstrated normocytic normochromic levels throughout the study period and as it did not have any serious impact nor did any patients complain of shortness of breath, this was ruled out to clinically non-significant. Hb levels were too high to be associated with fatigue.

HCV is associated with deranged LFT’s [12]. At presentation, most patients had pathological elevations of Alanine Transaminase (ALT), Aspartate Aminotransferase (AST), Alkaline Phosphatase (ALK-P), Gamma-Glutamyl Transferase (GGT). This would be expected in active HCV infection. With viral elimination, all LFTs improved over time. Lowest values were seen at 24 weeks since beginning treatment. Electrolyte, urea and creatinine profiles remained unchanged during the duration of the study.

## Conclusions

SOF in combination with RIB has great efficacy and safety for treatment of chronic HCV. The regimen is associated with high viral eradication rates and improvement in the liver biochemical profile with very few adverse events. It is superior to all previous medications used for HCV. SOF + RIB should now be recommended for the treatment of HCV until superseded by a newer and better regimen.

## Appendices

Shortcomings of the study:

The authors would like to acknowledge the following aspects that were beyond their control:

1. At the time of study in Pakistan, this drug regimen (SOF + RIB) was the only one licensed in the country; as such other superior regimen could not be given.

2. Transient elastography could not be done in all patients to assess fibrosis stage due to lack of availability.

3. Similarly, testing for IL28 B genotyping was unavailable and not carried out.

4. Patients were followed up until one year after starting treatment, post one year SVR or reactivation of the virus were not recorded after that. Efficacy of the drug cannot be stated beyond that point.

5. Conditions such as NAFLD and metabolic syndrome were not taken into account for analysis.
